# Detecting ADRD Caregivers’ Information Wants in Social Media: A Machine Learning–Aided Approach

**DOI:** 10.1093/geroni/igaa057.2261

**Published:** 2020-12-16

**Authors:** Bo Xie, Zhendong Wang, Ning Zou, Zhimeng Luo, Robin Hilsabeck, Alyssa Aguirre

**Affiliations:** 1 The University of Texas at Austin, Austin, Texas, United States; 2 University of Pittsburgh, Pittsburgh, Pennsylvania, United States; 3 University of Texas, Austin, Texas, United States; 4 Dell Medical School - University of Texas, Austin, Texas, United States

## Abstract

ADRD caregivers increasingly use social media to meet their health information wants (HIW). Machine learning (ML) tools may help understand caregivers’ HIW as expressed via social media. This pilot study explored a collaborative, iterative process between domain experts and ML tools to identify ADRD caregivers’ HIW from social media data. The HIW-ADRD framework was adapted from an existing HIW framework. Through multiple rounds of iteration between the experts and the ML tools, the framework was expanded to include 11 types of health information. Each type included corresponding keywords developed through a hybrid approach that included keywords from both the theoretical constructs (top-down) and caregivers’ posts (bottom-up). These keywords were then used to enhance the ML tools’ ability to code 106 recent posts extracted from an ADRD social media group in March 2020. When compared with expert coding results, ML tools accurately predicted 56% of HIW. Further work is underway.

